# A backstepping control of artificial pancreas for type 1 diabetes based on sub-fixed-time stability

**DOI:** 10.3389/fendo.2026.1729453

**Published:** 2026-02-26

**Authors:** Yuexian Xing, Hanjie Ma, Yongbo Zhang, Boyan Jiang, Kaiming Luo, Fei Hua

**Affiliations:** 1 Department of Endocrinology, The Third Affiliated Hospital of Soochow University, Changzhou, Jiangsu, China; 2 School of Information Science & Technology, Southwest Jiaotong University, Chengdu, Sichuan, China; 3 School of Electrical and Information Engineering, Changzhou Institute of Technology, Changzhou, Jiangsu, China

**Keywords:** artificial pancreas control, blood glucose regulation, power exponent control, sub-fixed-time stability, type 1 diabetes

## Abstract

**Introduction:**

The artificial pancreas device is an automated control system that simulates the function of the human pancreas. It continuously infuses insulin into the body, thereby maintaining the blood glucose levels of diabetic patients within a safe range. This device is expected to be widely adopted for patients with type 1 diabetes in the future. Currently, research on artificial pancreas control methods is still in its early stages. Most existing blood glucose control methods rely on controller designs that incorporate only gain parameters and typically lack rigorous theoretical analysis of closed-loop system stability. In contrast, the Power Exponent Controller (PEC), which introduces power exponent parameters, belongs to the categories of finite-time or fixed-time control. These controllers often demonstrate superior overall performance in terms of convergence rate, robustness, and other critical control metrics.

**Methods:**

This paper proposes an insulin infusion rate based on PEC. A comprehensive stability analysis of the blood glucose closed-loop system is conducted using backstepping control theory, particularly providing mathematical expressions for system convergence time and steady-state error. The proposed control method is evaluated through three sets of simulation experiments comparing it with a traditional homogeneous control method.

**Results:**

The theoretical findings suggest that the proposed control method effectively reduces disturbances caused by meals and the infusion process, allowing quick adjustment of the patient's blood glucose to the target range. The results from the three sets of simulation experiments demonstrate that, compared to the traditional homogeneous control method, the proposed PEC scheme offers several advantages: a faster and more responsive reduction in hyperglycemia; the ability to consistently maintain postprandial glucose peaks below 180 mg/dL despite glucose fluctuations caused by three daily meals; and a reduction of approximately 25 minutes in the time required to bring blood glucose into the safe range during extreme daily regulation scenarios involving initial hyperglycemia.

**Discussion:**

These findings indicate that the proposed PEC method provides improved performance for artificial pancreas systems, with potential benefits for clinical management of type 1 diabetes.

## 1 Introduction

Diabetes is a chronic metabolic disease characterized by hyperglycemia and is one of the most prevalent chronic diseases worldwide. In 2021, approximately 10.5% of adults were diagnosed with diabetes, and the global prevalence is projected to increase to 12.2% by 2045 (1). It is reported that diabetes accounts for one in every nine adult deaths (2).

Diabetes is primarily classified into two types: Type 1 and Type 2. Type 1 diabetes is a chronic, progressive autoimmune disease characterized by the irreversible destruction of insulin-producing beta cells in the pancreas. Due to the absolute deficiency of insulin, individuals with Type 1 diabetes often rely on exogenous insulin to decrease plasma glucose, and they often experience significant and unpredictable glucose fluctuations (3). This abnormal fluctuation in plasma glucose can trigger a series of complications within the human body, including cardiovascular diseases, diabetic retinopathy, diabetic nephropathy, diabetic peripheral neuropathy, and diabetic foot, among others, posing significant challenges to quality of life (4, 5),. Compared with type 2 diabetes, patients with type 1 diabetes have a higher risk of cardiovascular events and all-cause mortality (6). Therefore, optimizing blood glucose management for patients with type 1 diabetes is crucial.

There are two main methods for administering exogenous insulin. One common approach is the insulin pen, which requires patients to manually inject insulin subcutaneously multiple times a day—a process that can be cumbersome. Another widely used method is the insulin pump, which automatically and continuously delivers insulin subcutaneously into the body. Compared to insulin pens, insulin pumps offer greater flexibility, ease of use, and reduced discomfort during administration. They also enable more precise, stable, and continuous blood glucose control (7, 8). Due to differences in control mechanisms, two common types of insulin pump delivery systems are available on the market. The first is the open-loop system, where the physician determines the patient’s basal insulin dose and mealtime bolus doses based on clinical experience. This delivery method requires patients to follow a strict schedule for meals and maintain a highly regular lifestyle. During exercise or in stressful situations, significant fluctuations in blood glucose are inevitable and can potentially become life-threatening in severe cases. The other type of insulin pump delivery system is the closed-loop system. A closed-loop insulin delivery system consists of three components: a continuous glucose monitor (CGM), an insulin pump, and a control algorithm that determines the exogenous insulin delivery rate (IDR). This system is also known as an artificial pancreas (9).

Compared to open-loop insulin pumps, the core advantage of closed-loop systems lies in their intelligent control algorithm, which automatically adjusts the insulin infusion rate based on real-time glucose monitoring data, thereby effectively reducing the occurrence of hyperglycemic and hypoglycemic events (10, 11). Therefore, the closed-loop control algorithm serves as the core of the artificial pancreas, playing a decisive role in blood glucose regulation. However, in clinical practice, we observe significant variability among individuals or even within the same individual over time due to differences or changes in diet, exercise, daily routine, illness, and stress levels. These uncertainties collectively contribute to the suboptimal performance of closed-loop insulin pumps in achieving stable glycemic control. To overcome these challenges and realize optimal glucose management, it is essential to appropriately design the insulin delivery rate control algorithm. This endeavor is not only highly practical but also holds substantial promise.

Over the past decade, continuous glucose monitoring systems (CGMS) have achieved technological breakthroughs and have been widely commercialized. These developments have established the foundation for the design of closed-loop glucose control systems, making their development into a reality. As CGMS technology advances, more researchers are concentrating on developing closed-loop glucose control algorithms. For example, Refs (12, 13) designed blood glucose control algorithms based on model predictive control methods; Ref (14) designed blood glucose control algorithms based on LPV control methods; Refs (15, 16) developed a blood glucose regulation algorithm utilizing the PID control approach; and Refs (17, 18) designed a blood glucose control algorithm based on sliding mode control methods. In recent years, several researchers have increasingly acknowledged the significance of performing stability theoretical analysis on closed-loop blood glucose control systems. For example, Refs (19–22), and (23, 24), respectively use model predictive control, fuzzy control, sliding mode control, and backstepping control methods to design insulin infusion rates and analyze the stability of the system.

The control methods presented in Refs (12–24) are based on controller designs that employ only gain parameters and do not incorporate power exponent parameters. The appropriate introduction of power exponent parameters into a controller can often enhance a system’s control performance. Based on the stability characteristics of the closed-loop system, such power exponent control is referred to as finite-time control if it achieves finite-time stability, or fixed-time control if it achieves fixed-time stability. For consistency, this paper collectively terms this control method incorporating power exponent parameters as Power Exponent Control (PEC). Ref (25) uses mathematical derivation to demonstrate that PEC possesses faster convergence speed, higher precision, and better disturbance rejection capabilities compared to linear control. Ref (26) offers a finer classification of finite-time control within the PEC framework, dividing it into twenty-three specific control methods and discussing the advantages and disadvantages of each in detail. Although PEC has shown superior performance in many control fields, its research in the domain of the artificial pancreas remains relatively limited. For example, in our prior work [Ref (27)], we investigated the preliminary application of homogeneous control (a classic PEC method) to the artificial pancreas. However, as a fundamental type of power exponent control, homogeneous control has certain limitations in terms of robustness and disturbance rejection. Given that the human blood glucose regulation process is susceptible to various uncertainties and disturbances, and considering the critical importance of achieving rapid and stable glucose reduction to mitigate the risks of hyperglycemia, further research on PEC methods with stronger robustness, suitable for artificial pancreas scenarios, holds clear academic value and clinical significance. This constitutes the research motivation and core objective of this paper.

This paper addresses two primary issues noted above: the scarcity of stability analysis results for current artificial pancreas control systems and the limited research on PEC specific to this domain. It investigates the PEC design methodology for artificial pancreas control systems and conducts a thorough stability analysis of the corresponding closed-loop system under disturbances. The main contributions and research objectives of this paper are outlined as follows.

1. For the automatic blood glucose regulation system in type 1 diabetic patients managed by an artificial pancreas, the insulin infusion rate is designed using backstepping control and the PEC theory. Dual power exponent parameters are incorporated into the control algorithm to achieve satisfactory control performance for blood glucose both near and away from the equilibrium point.
2. Beyond accounting for meal-induced glucose disturbances, the Bergman Minimal Model (BMM) used in this paper explicitly incorporates disturbances arising from various internal and external uncertainties during the insulin infusion process. The disturbance rejection capability of the proposed controller against both types of disturbances is rigorously analyzed.
3. Utilizing the Lyapunov method, explicit mathematical relationships are established among the steady-state blood glucose error, system convergence time, and key control and model parameters. This provides robust theoretical guarantees for the effectiveness of the designed closed-loop system in achieving safe and efficient glucose regulation.

Furthermore, it is worth noting that the PEC studied in this paper falls within the realm of non-smooth control theory. Compared to another method commonly used in diabetes treatment, Multiple Daily Injections (MDI), both can be broadly defined as non-smooth control methods in a mathematical sense. However, there exists an essential distinction between them in terms of design principles and application scenarios. The MDI method is a typical impulsive control at the algorithmic design level. It primarily achieves blood glucose regulation through optimal dose injections at sparse time points and is particularly suitable for resource-constrained scenarios or for patients who decline continuous monitoring devices, such as CGM. It has become an important research direction in diabetes treatment. For example, Borri et al. (28) simplified online computation using a periodic strategy, while Mirzaee et al. (29) enhanced the ability to handle uncertainties through global optimization. In contrast, a key point of differentiation between the PEC studied in this paper and MDI lies in the theoretical foundation: the PEC in this work is a continuous control strategy, not an impulsive one. Therefore, although impulsive control plays an indispensable role in MDI schemes, the control method developed in this paper is not designed for the MDI scheme. Instead, it is more suitable for real-time, precise blood glucose regulation in scenarios that involve high-precision continuous monitoring and infusion.

The remaining sections of this paper are organized as follows. Section 2 presents the primary innovative contributions, introducing the blood glucose control model and control objectives for patients with type 1 diabetes. Building on this foundation, it proposes the PEC method designed in this study and details the theoretical analysis of its stability. Section 3 designs three simulation scenarios with different initial blood glucose conditions for diabetic patients. Using MATLAB simulation software and patient parameters from the UVA/Padova T1DM simulator, it conducts simulation experiments to evaluate the glucose-lowering efficacy of the proposed PEC scheme. Section 4 provides a detailed comparison and analysis of the glucose-lowering effectiveness between the PEC scheme developed here and the PEC scheme from Ref (27). It further discusses the superior performance of the controller designed in this paper and the underlying reasons. Finally, Section 5 presents the conclusion.

## 2 Model and control method

This section primarily introduces the mathematical models, control objectives, and control algorithms used in the study of automatic blood glucose control systems.

### 2.1 Mathematical model

Among the current research findings on blood glucose control, the most widely used mathematical model to describe the relationship between insulin and blood glucose is the Bergman’s minimal model (30, 31),which is expressed as follows:

(1) $$\left\{ \begin{array}{l} \dot{G}=-{\text{c}}_{1}(G-{\text{G}}_{\text{b}})-GX+w_{1}\\ \dot{X}=-{\text{c}}_{2}X+{\text{c}}_{3}(I-{\text{I}}_{\text{b}})\\ \dot{I}=-{\text{c}}_{4}(I-{\text{I}}_{\text{b}})+{\text{c}}_{5}u+w_{2} \end{array}\right.$$

where G represents the blood glucose concentration, measured in mg/dL, ${\text{G}}_{\text{b}}$ denotes the baseline blood glucose value, also in mg/dL, X is the glucose-lowering effect of insulin, expressed in min^-1^, I represents the insulin concentration, measured in mU/L, ${\text{I}}_{\text{b}}$is the baseline insulin value, in mU/L, u denotes the insulin infusion rate, which also the control algorithm to be designed, measured in mU/min, $c_{1}$ is the rate coefficient for glucose transport from plasma space into the liver or peripheral tissues, expressed in min^-1^, $c_{2}$ is the rate coefficient for the reduction of insulin’s glucose-lowering effect, in min^-1^, $c_{3}$ represents the rate coefficient of plasma insulin acting on insulin’s glucose-lowering effect, with units of L·mU^-1^·min^-2^, $c_{4}$ is the insulin decay rate coefficient, in min^-1^, $c_{5}$ denotes the reciprocal of the insulin distribution volume, measured in L^-1^. $w_{1}=w_{11}+w_{12}$ denotes the disturbance affecting the rate of blood glucose fluctuation, while $w_{2}=w_{21}+w_{21}$ denotes the disturbance affecting the rate of insulin concentration change. $w_{11}$ represents the fluctuations in the rate of blood glucose concentration caused by diet. $w_{12}$represents the uncertainty in blood glucose concentration caused by unmodeled factors. $w_{21}$ represents disturbances to the insulin infusion rate caused by various internal and external uncertainties during the infusion process, such as errors arising from the discretization of continuous insulin delivery rates in practical applications. $w_{22}=\text{sat}(u)-u$ can be interpreted as the error between ideal continuous control u and the real insulin delivery, where saturation function, sat(·), is used to constrain the insulin infusion rate, with a lower limit of 0 and an upper limit typically on the order of tens of mU/min. It is worth mentioning that, the insulin infusion disturbance term $w_{2}$ introduced in model (1) has not been adequately addressed in existing stability analyses of closed-loop glucose control. Although its inclusion complicates the subsequent stability analysis, it substantially improves the model’s engineering fidelity, thereby allowing for a more effective validation of the disturbance rejection capability of the designed control method.

*Remark 1*: The Bergman’s minimal model (1) adopted in this paper, if applied directly to a practical artificial pancreas system, has certain limitations. This model assumes that insulin is infused directly into the plasma and does not account for the significant absorption delay following subcutaneous insulin infusion. We employ this model in our study for two primary reasons. First, it is the most widely used and foundational model in artificial pancreas control research. Second, its simplified nature allows for clear theoretical derivation and stability proof. This paper aims to introduce power exponent control into the blood glucose regulation problem and to demonstrate that the closed-loop system can achieve sub-fixed-time stability. It should be noted that, because the model does not incorporate subcutaneous absorption dynamics, the finite convergence time derived for the closed-loop system in this paper is theoretically shorter than the time required for blood glucose to reach the target range in real clinical scenarios. Therefore, future work will need to consider models that include time delays or higher-order dynamics. The controller design and stability analysis for such models will be an important direction for future research.

### 2.2 Control method and control objective

#### 2.2.1 Control method

The system state variables in the blood glucose model (1) are G, X and I. Further, the state error variables are defined as $\epsilon_{\text{G}}≜G-$ ${\text{G}}_{\text{d}}$, $\epsilon_{\text{X}}≜X-X_{\text{d}}$and $\epsilon_{\text{I}}≜I-I_{\text{d}}$, where ${\text{G}}_{\text{d}}$ is a constant representing the target blood glucose concentration, $X_{\text{d}}$ and $I_{\text{d}}$ are virtual control laws to be designed. To achieve blood glucose sub-fixed-time stabilization, this paper proposes the following insulin infusion rate u and virtual control laws $X_{\text{d}}$ and $I_{\text{d}}$:

(2) $$u=-{\text{k}}_{4}\text{sig}{\left(I-I_{\text{d}}\right)}^{{\text{p}}_{1}}-{\text{c}}_{5}^{-1}m$$

(3) $$I_{\text{d}}≜-{\text{k}}_{3}{\text{c}}_{3}^{-1}\epsilon_{\text{X}}+{\text{c}}_{2}{\text{c}}_{3}^{-1}X+{\text{c}}_{3}^{-1}m_{1}l+{\text{I}}_{\text{b}}$$

(4) $$X_{\text{d}}≜{\text{k}}_{1}\text{sig}{\left(\epsilon_{\text{G}}\right)}^{{\text{g}}_{1}}G^{-1}+{\text{k}}_{2}\epsilon_{\text{G}}G^{-1}-{\text{c}}_{1}(G-{\text{G}}_{\text{b}})G^{-1}$$

where, the constants ${\text{k}}_{1}$, ${\text{k}}_{2}$, ${\text{k}}_{3}$ and ${\text{k}}_{4}$ are control gain parameters. Constants ${\text{g}}_{1}$ and ${\text{p}}_{1}$ represent power exponent control parameters used to further enhance control performance, and they satisfy ${\text{g}}_{1}>1$and ${\text{p}}_{1}\in (0,1)$, with function $\text{sig}{\left(\epsilon_{\text{I}}\right)}^{{\text{p}}_{1}}$ representing $\text{sign}(\epsilon_{\text{I}})|\epsilon_{\text{I}}{|}^{{\text{p}}_{1}}$. The definitions of variables m, l and $m_{1}$ are as follows:


$$m≜-{\text{c}}_{4}(I-{\text{I}}_{\text{b}})-m_{5}$$

$$l≜-{\text{c}}_{1}(G-{\text{G}}_{\text{b}})-GX$$

$$\begin{array}{l} m_{1}≜{\text{k}}_{1}{\text{g}}_{1}|\epsilon_{\text{G}}{|}^{{\text{g}}_{1}-1}G^{-1}-{\text{k}}_{1}\text{sig}(\epsilon_{\text{G}}){\text{g}}_{1}G^{-2}+{\text{k}}_{2}G^{-1}-\\ \;\;\;\;\;\;\;{\text{k}}_{2}\epsilon_{\text{G}}G^{-2}-{\text{c}}_{1}{\text{G}}_{\text{b}}G^{-2} \end{array}$$

$$\begin{array}{l} m_{2}≜-{\text{k}}_{1}{\text{g}}_{1}\text{sig}{(}^{\varepsilon_{\text{G}}}{)}^{{\text{g}}_{1}-1}G^{-2}+{\text{k}}_{1}{\text{g}}_{1}({\text{g}}_{1}-1)\text{sig}(\varepsilon_{\text{G}}){\text{g}}_{1}-2G^{-1}+\\ \;\;\;\;\;\;2{\text{k}}_{1}\text{sig}{(}^{\varepsilon_{\text{G}}}{)}^{{\text{g}}_{1}}G^{-3}-{\text{k}}_{1}{\text{g}}_{1}|\varepsilon_{\text{G}}{|}^{{\text{g}}_{1}-1}G^{-1} \end{array}$$

$$m_{3}≜m_{2}-{\text{k}}_{2}G^{-2}-{\text{k}}_{2}G^{-2}+2{\text{k}}_{2}\varepsilon_{\text{G}}G^{-3}$$

$$m_{4}≜-{\text{c}}_{2}X+{\text{c}}_{3}(I-{\text{I}}_{\text{b}})-m_{1}l$$

$$\begin{array}{l} m_{5}≜{\text{c}}_{3}^{-1}m_{3}l^{2}+{\text{c}}_{1}{\text{c}}_{3}^{-1}{\text{G}}_{\text{b}}G^{-2}l({\text{c}}_{1}l+Xl+G\dot{X})-{\text{k}}_{3}{\text{c}}_{3}^{-1}m_{4}+\\ \;\;\;\;2{\text{c}}_{1}{\text{c}}_{3}^{-1}{\text{G}}_{\text{b}}G^{-3}l^{3}+{\text{c}}_{2}{\text{c}}_{3}^{-1}\dot{X}-{\text{c}}_{3}^{-1}m_{1}({\text{c}}_{1}+X)l-{\text{c}}_{3}^{-1}m_{1}G\dot{X} \end{array}$$

#### 2.2.2 Control objective

**Figure 1:**
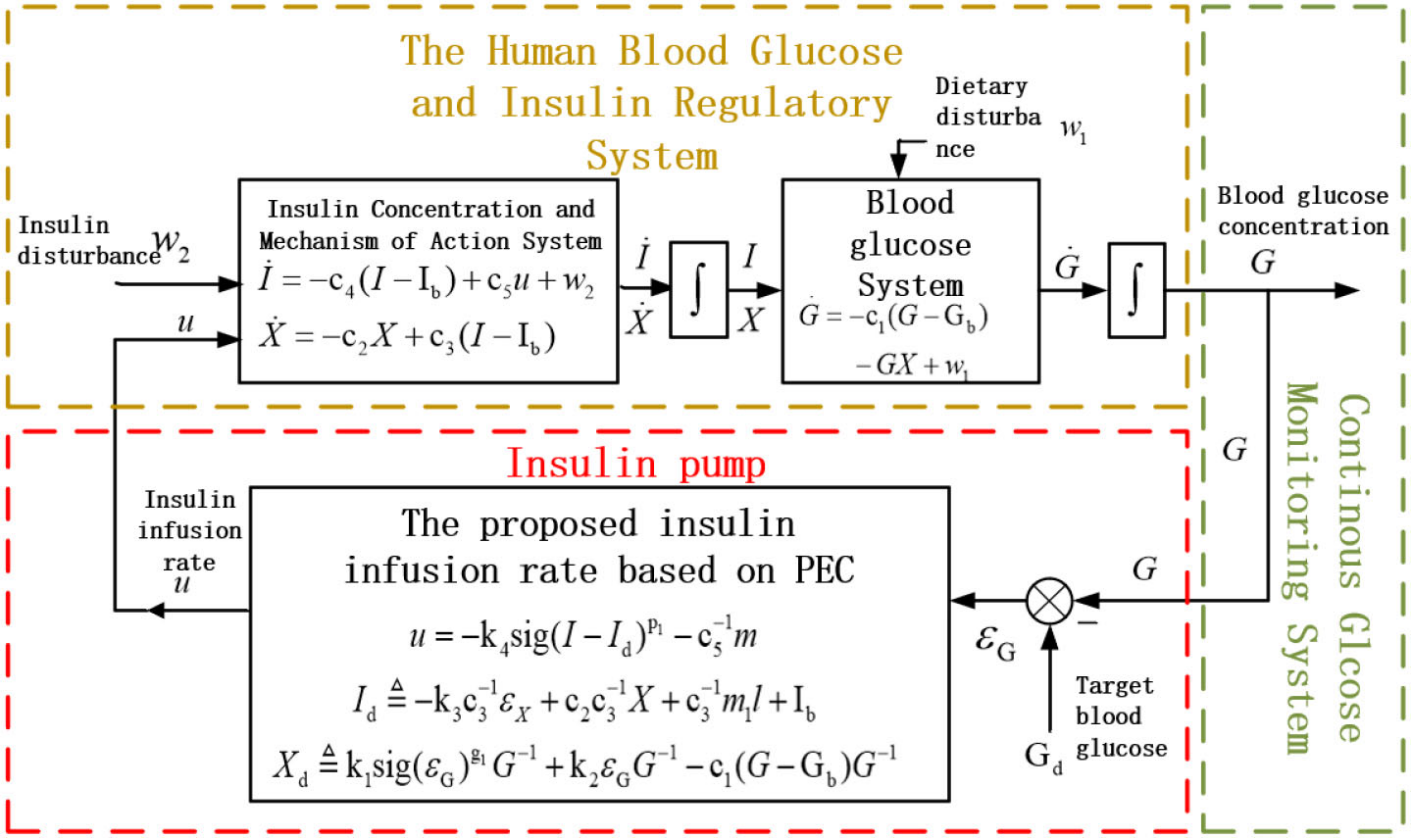
Schematic diagram of closed-loop blood glucose control for artificial pancreas.

**Figure 1** illustrates the relationship between the proposed controller (2)-(4) and the previously mentioned blood glucose model (1) and control objective ${\text{G}}_{\text{d}}$. As shown in **Figure 1**, there are three external inputs to the closed-loop system: the target blood glucose ${\text{G}}_{\text{d}}$, blood glucose fluctuations caused by diet $w_{1}$, and the unmodeled uncertainties in the insulin infusion process $w_{2}$. The insulin pump automatically delivers insulin to the body based on the insulin infusion rate u The patient’s blood glucose G changes in real time under the influence of $w_{1}$, $w_{2}$ and u. The CGM feeds the real-time observed blood glucose G back to the control system u to form a closed loop. Based on the principle of this closed-loop control, the main control objective of this paper is to design insulin infusion rate u (2)-(4), so that the patient’s blood glucose G, despite being subjected to dual disturbances from both $w_{1}$ and $w_{2}$, can still converge to a small neighborhood of ${\text{G}}_{\text{d}}$—that is, the error variable $\epsilon_{\text{G}}$ converges to a small range near 0.

#### 2.2.3 Stability analysis methods

This section aims to provide a theoretical analysis of the dynamic performance of blood glucose concentration as it converges to its target value under the influence of the proposed controller acting on the glucose model. The analysis specifically focuses on determining the upper bounds for both the convergence time and the steady-state error. To complete this analysis, it is necessary to proceed in the following three steps, examining the convergence of the insulin tracking error $\epsilon_{\text{I}}$, the remote insulin effect tracking error $\epsilon_{\text{X}}$, and the blood glucose tracking error $\epsilon_{\text{G}}$, respectively.

Step 1: Convergence Analysis of $\epsilon_{\text{I}}$.

Define the Lyapunov candidate function $V_{\text{I}}≜\epsilon_{\text{I}}^{2}$, whose derivative is ${\dot{V}}_{\text{I}}=2\epsilon_{\text{I}}{\dot{\epsilon }}_{\text{I}}=2\epsilon_{\text{I}}\dot{I}-2\epsilon_{\text{I}}{\dot{I}}_{\text{d}}$ Taking the derivative of $I_{\text{d}}$ in Equation 3 yields.

(5) $${\dot{I}}_{\text{d}}={\text{c}}_{3}^{-1}l{\dot{m}}_{1}-{\text{k}}_{3}{\text{c}}_{3}^{-1}{\dot{\epsilon }}_{\text{X}}+{\text{c}}_{2}{\text{c}}_{3}^{-1}\dot{X}+{\text{c}}_{3}^{-1}m_{1}\dot{l}$$

Calculate the values of ${\dot{m}}_{1}$, $\ddot{G}$, ${\dot{\epsilon }}_{\text{X}}$ and $\dot{l}$ separately as follows


$${\dot{m}}_{1}=m_{3}\dot{G}+2{\text{c}}_{1}{\text{G}}_{\text{b}}G^{-3}{\dot{G}}^{2}-{\text{c}}_{1}{\text{G}}_{\text{b}}G^{-2}\ddot{G}$$

$$\ddot{G}=-({\text{c}}_{1}+X)l-G\dot{X}-({\text{c}}_{1}+X)w_{1}+{\dot{w}}_{1}$$

$${\dot{\epsilon }}_{\text{X}}=m_{4}-m_{1}w_{1}$$

$$\dot{l}=-({\text{c}}_{1}+X)l-G\dot{X}-({\text{c}}_{1}+X)w_{1}$$

By substituting the above four equations into Equation 5 and simplifying, we can obtain the value of ${\dot{I}}_{\text{d}}$ as.

(6) $${\dot{I}}_{\text{d}}={\text{c}}_{3}^{-1}l{\dot{m}}_{1}-{\text{k}}_{3}{\text{c}}_{3}^{-1}{\dot{\epsilon }}_{\text{X}}+{\text{c}}_{2}{\text{c}}_{3}^{-1}\dot{X}+{\text{c}}_{3}^{-1}m_{1}\dot{l}$$

To further simplify the above expression, we need to define an auxiliary variable $m_{6}$ as follows:


$$\begin{array}{l} m_{6}≜{\text{c}}_{3}^{-1}m_{3}l+{\text{c}}_{1}{\text{c}}_{3}^{-1}{\text{G}}_{\text{b}}G^{-2}l({\text{c}}_{1}+X)+4{\text{c}}_{1}{\text{c}}_{3}^{-1}{\text{G}}_{\text{b}}G^{-3}l^{2}+\\ \;\;\;\;\;\;{\text{k}}_{3}{\text{c}}_{3}^{-1}m_{1}-{\text{c}}_{3}^{-1}m_{1}({\text{c}}_{1}+X) \end{array}$$

According to the definitions of $m_{5}$ and $m_{6}$ the above ${\dot{I}}_{\text{d}}$ in Equation 6 can be simplified as.

(7) $${\dot{I}}_{\text{d}}=m_{5}+m_{6}w_{1}+2{\text{c}}_{1}{\text{c}}_{3}^{-1}{\text{G}}_{\text{b}}G^{-3}lw_{1}^{2}-{\text{c}}_{1}{\text{c}}_{3}^{-1}{\text{G}}_{\text{b}}G^{-2}l{\dot{w}}_{1}$$

To simplify subsequent analysis, a comprehensive disturbance variable $w_{\text{I}}$ is defined as follows:

$w_{\text{I}}≜-m_{6}w_{1}-2{\text{c}}_{1}{\text{c}}_{3}^{-1}{\text{G}}_{\text{b}}G^{-3}lw_{1}^{2}+{\text{c}}_{1}{\text{c}}_{3}^{-1}{\text{G}}_{\text{b}}G^{-2}l{\dot{w}}_{1}$Substitute $\dot{I}$ in Equation 1 and ${\dot{I}}_{\text{d}}$ in Equation 7 into ${\dot{V}}_{\text{I}}=2\epsilon_{\text{I}}\dot{I}-2\epsilon_{\text{I}}{\dot{I}}_{\text{d}}$, and use the definition of $w_{\text{I}}$ to obtain.

(8) $${\dot{V}}_{\text{I}}=2{\text{c}}_{5}\epsilon_{\text{I}}u+2\epsilon_{\text{I}}m+2\epsilon_{\text{I}}w_{\text{I}}$$

Since a closed-loop system can only suppress bounded disturbances, it is reasonable and necessary to assume that the disturbance term $w_{\text{I}}$ is bounded, i.e., there exists a constant ${\bar{\text{w}}}_{\text{I}}$ such that $|w_{\text{I}}|\leq {\bar{\text{w}}}_{\text{I}}$.

Substituting the designed insulin infusion rate (2) into Equation 8 yields.


$${\dot{V}}_{\text{I}}=-2{\text{k}}_{4}{\text{c}}_{5}|\epsilon_{\text{I}}{|}^{1+{\text{p}}_{1}}+2\epsilon_{\text{I}}w_{\text{I}}$$

Bounding the last term gives.


$${\dot{V}}_{\text{I}}\leq -2{\text{k}}_{4}{\text{c}}_{5}|\epsilon_{\text{I}}{|}^{1+{\text{p}}_{1}}+2{\bar{w}}_{\text{I}}|\epsilon_{\text{I}}|$$

For an arbitrary constant ${\text{θ}}_{\text{I}}\in (0,1)$, we can further derive that.

(9) $$\begin{array}{l} {\dot{V}}_{\text{I}}\leq -2{\text{k}}_{4}{\text{c}}_{5}(1-{\text{θ}}_{\text{I}})|\epsilon_{\text{I}}{|}^{1+{\text{p}}_{1}}-\\ \;2{\text{θ}}_{\text{I}}{\text{k}}_{4}{\text{c}}_{5}|\epsilon_{\text{I}}{|}^{1+{\text{p}}_{1}}+2{\bar{w}}_{\text{I}}|\epsilon_{\text{I}}| \end{array}$$

Define the set ${\text{D}}_{\text{I}}≜\{\epsilon_{\text{I}}:|\epsilon_{\text{I}}|<\Delta_{\text{I}}\}$, where $\Delta_{\text{I}}≜$ $({\bar{w}}_{\text{I}}{\text{θ}}_{\text{I}}^{-1}$ ${\text{k}}_{4}^{-1}{\text{c}}_{5}^{-1}{)}^{{1/p}_{1}}$. Then, whenever $\epsilon_{\text{I}}\notin {\text{D}}_{\text{I}}$ (i.e., $|\epsilon_{\text{I}}|>\Delta_{\text{I}}$), Equation 9 implies.


$${\dot{V}}_{\text{I}}\leq -2{\text{k}}_{4}{\text{c}}_{5}(1-{\text{θ}}_{\text{I}})V_{\text{I}}^{(1+{\text{p}}_{1})/2}$$

By Lemma 1, the error $\epsilon_{\text{I}}$ converges to the set ${\text{D}}_{\text{I}}$ in a finite time ${\text{T}}_{\text{I}}$, whose upper bound is given by.


$${\text{T}}_{\text{I}}\leq {\text{k}}_{4}^{-1}{\text{c}}_{5}^{-1}{\left(1-{\text{θ}}_{\text{I}}\right)}^{-1}{\left(1-{\text{p}}_{1}\right)}^{-1}V_{\text{I}}{\left(\epsilon_{I0}\right)}^{(1-{\text{p}}_{1})/2}$$

where $\epsilon_{\text{I}0}$ is the initial value of $\epsilon_{\text{I}}$.

Step 2: Convergence Analysis of $\epsilon_{\text{X}}$.

Define the Lyapunov candidate function $V_{\text{X}}≜\epsilon_{\text{X}}^{2}$, whose derivative is ${\dot{V}}_{\text{X}}=2\epsilon_{\text{X}}{\dot{\epsilon }}_{\text{X}}$. Taking the derivative of $X_{\text{d}}$ in Equation 4 yields.


$$\begin{array}{l} {\dot{X}}_{\text{d}}=[{\text{k}}_{1}{\text{g}}_{1}|\epsilon_{\text{G}}{|}^{{\text{g}}_{1}-1}G^{-1}-{\text{k}}_{1}\text{sig}{(}^{\epsilon_{\text{G}}}{)}^{{\text{g}}_{1}}G^{-2}+\\ {\text{k}}_{2}G^{-1}-{\text{k}}_{2}\epsilon_{\text{G}}G^{-2}-{\text{c}}_{1}{\text{G}}_{\text{b}}G^{-2}](l+w_{1}) \end{array}$$

Using the definition of $m_{1}$, the above expression can be simplified to ${\dot{X}}_{\text{d}}=m_{1}l+m_{1}w_{1}$. Substituting this and $\dot{X}$ in Equation 1 into ${\dot{\epsilon }}_{\text{X}}=\dot{X}-{\dot{X}}_{\text{d}}$ yields.

(10) $${\dot{\epsilon }}_{\text{X}}=-{\text{c}}_{2}X+{\text{c}}_{3}(I-{\text{I}}_{\text{b}})-m_{1}l-m_{1}w_{1}$$

Note that $I=I_{\text{d}}+\epsilon_{\text{I}}$. By substituting the definition of $I_{\text{d}}$ into $I=I_{\text{d}}+\epsilon_{\text{I}}$, we get.


$$I=-{\text{k}}_{3}{\text{c}}_{3}^{-1}\epsilon_{\text{X}}+{\text{c}}_{2}{\text{c}}_{3}^{-1}X+{\text{c}}_{3}^{-1}m_{1}l+{\text{I}}_{\text{b}}+\epsilon_{\text{I}}$$

Substituting the above equation into Equation 10, we get.

(11) $${\dot{\epsilon }}_{\text{X}}=-{\text{k}}_{3}\epsilon_{\text{X}}-w_{\text{X}}+{\text{c}}_{3}\epsilon_{\text{I}}$$

where $w_{\text{X}}≜m_{1}w_{1}$ represents the composite disturbance term. Substituting (Equation 11) from the above equation into ${\dot{V}}_{\text{X}}=2\epsilon_{\text{X}}{\dot{\epsilon }}_{\text{X}}$ yields.

(12) $${\dot{V}}_{\text{X}}=-2{\text{k}}_{3}\epsilon_{\text{X}}\epsilon_{\text{X}}-2\epsilon_{\text{X}}w_{\text{X}}+2{\text{c}}_{3}\epsilon_{\text{X}}\epsilon_{\text{I}}$$

The analysis in the previous section shows that the error $\epsilon_{\text{I}}$ converges to and remains within the set ${\text{D}}_{\text{I}}$ after a finite time $T_{\text{I}}$. Hence, for $t\geq {\text{T}}_{\text{I}}$, we have $|\epsilon_{\text{I}}|\leq \Delta_{\text{I}}$. Let ${\bar{w}}_{\text{X}}$ denote the upper bound of the disturbance term $w_{\text{X}}$. Then, from Equation 12, we obtain the following inequality:


$${\dot{V}}_{\text{X}}\leq -2{\text{k}}_{3}\epsilon_{\text{X}}^{2}+2|\epsilon_{\text{X}}{|\bar{w}}_{\text{X}}+2{\text{c}}_{3}|\epsilon_{\text{X}}|\Delta_{\text{I}}$$

For an arbitrary constant ${\text{θ}}_{\text{X}}\in (0,1)$, this can be rewritten as.

(13) $$\begin{array}{l} {\dot{V}}_{\text{X}}\leq -2{\text{k}}_{3}(1-{\text{θ}}_{\text{X}})\epsilon_{\text{X}}^{2}-2{\text{k}}_{3}{\text{θ}}_{\text{X}}\epsilon_{\text{X}}^{2}+\\ \;2|\epsilon_{\text{X}}|{\bar{\text{w}}}_{\text{X}}+2{\text{c}}_{3}|\epsilon_{\text{X}}|{\text{Δ}}_{\text{I}} \end{array}$$

Define the set ${\text{D}}_{\text{X}}≜\{\epsilon_{\text{X}}:|\epsilon_{\text{X}}|<{\text{Δ}}_{\text{X}}\}$with ${\text{Δ}}_{\text{X}}≜{\text{θ}}_{\text{X}}^{-1}{\text{k}}_{3}^{-1}$ $\left({\text{c}}_{3}{\text{Δ}}_{\text{I}}+{\bar{\text{w}}}_{\text{X}}\right)$. Whenever $\epsilon_{\text{X}}\notin {\text{D}}_{\text{X}}$ (i.e., $|\epsilon_{\text{X}}|>{\text{Δ}}_{\text{X}}$), inequality (Equation 13) implies that.

(14) $${\dot{V}}_{\text{X}}\leq -2{\text{k}}_{3}(1-{\text{θ}}_{\text{X}})V_{\text{X}}$$

always holds. This shows that $\epsilon_{\text{X}}$ will converge and stabilize within the set ${\text{D}}_{\text{X}}$. Definition $\epsilon_{\text{X}}$ as the time from moment t=0 until final convergence to the set ${\text{D}}_{\text{X}}$ denoted as ${\text{T}}_{\text{X}}$. Below, we calculate the upper bound of the convergence time ${\text{T}}_{\text{X}}$. By integrating both sides of inequality (14), we obtain.

(15) $$\int_{V_{\text{X}}({\text{T}}_{\text{I}})}^{V_{\text{X}}({\text{T}}_{\text{x}})}V_{\text{X}}^{-1}\text{d}V_{\text{X}}\leq \int_{{\text{T}}_{\text{I}}}^{{\text{T}}_{\text{x}}}-2{\text{k}}_{3}(1-{\text{θ}}_{\text{X}})\text{d}t$$

where $V_{\text{X}}({\text{T}}_{\text{x}})$ represents the value corresponding to $V_{\text{X}}$ at $t{\text{=T}}_{\text{X}}$, and $V_{\text{X}}({\text{T}}_{\text{I}})$ represents the value corresponding to $V_{\text{X}}$ at $t{\text{=T}}_{\text{I}}$. By calculating the definite integral inequality (Equation 15), we obtain.


$$\ln V_{\text{X}}|\begin{array}{l} V_{\text{X}}({\text{T}}_{\text{x}})\\ V_{\text{X}}({\text{T}}_{\text{I}}) \end{array}\leq -2{\text{k}}_{3}(1-{\text{θ}}_{\text{X}})|\begin{array}{l} {\text{T}}_{\text{x}}\\ {\text{T}}_{\text{I}} \end{array}$$

Further simplification of the above equation yields:

(16) $$\ln V_{\text{X}}({\text{T}}_{\text{x}})-\ln V_{\text{X}}({\text{T}}_{\text{I}})\leq -2{\text{k}}_{3}(1-{\text{θ}}_{\text{X}})({\text{T}}_{\text{x}}-{\text{T}}_{\text{I}})$$

Note that $V_{\text{X}}({\text{T}}_{\text{x}})={\text{Δ}}_{\text{X}}^{2}$ and $V_{\text{X}}({\text{T}}_{\text{I}})=\epsilon_{\text{X}}{{\text{(T}}_{\text{I}})}^{2}$, then by further rearranging Equation 16, the upper bound of the convergence time ${\text{T}}_{\text{X}}$ can be obtained as.

(17) $${\text{T}}_{\text{x}}\leq {\text{T}}_{\text{I}}+{\text{k}}_{3}^{-1}{\left(1-{\text{θ}}_{\text{X}}\right)}^{-1}(\ln\epsilon_{\text{X}}{\text{(T}}_{\text{I}})-\ln\Delta_{\text{X}})$$

Step 3: Convergence Analysis of $\epsilon_{\text{G}}$.

Define the Lyapunov candidate function $V_{\text{G}}≜\epsilon_{\text{G}}^{2}$, and use the expression of $\dot{G}$ in Equation 1 to calculate the derivative of ${\dot{\epsilon }}_{\text{G}}$ as follows.

(18) $${\dot{\epsilon }}_{\text{G}}=\dot{G}-{\dot{G}}_{\text{d}}=-{\text{c}}_{1}(G-{\text{G}}_{\text{b}})-GX+w_{1}$$

Substituting the definition of $X_{\text{d}}$ in Equation 4 into $X=X_{\text{d}}+\epsilon_{\text{X}}$ yields.


$$X={\text{k}}_{1}\text{sig}{\left(\epsilon_{\text{G}}\right)}^{{\text{g}}_{1}}G^{-1}+{\text{k}}_{2}\epsilon_{\text{G}}G^{-1}-{\text{c}}_{1}(G-{\text{G}}_{\text{b}})G^{-1}+\epsilon_{\text{X}}$$

By substituting the above equation into Equation 18, we get.


$${\dot{\epsilon }}_{\text{G}}=-{\text{k}}_{1}\text{sig}{\left(\epsilon_{\text{G}}\right)}^{{\text{g}}_{1}}-{\text{k}}_{2}\epsilon_{\text{G}}+w_{1}+\epsilon_{\text{X}}$$

Substituting the above equation into ${\dot{V}}_{\text{G}}=2\epsilon_{\text{G}}{\dot{\epsilon }}_{\text{G}}$ yields.

(19) $${\dot{V}}_{\text{G}}=-2\epsilon_{\text{G}}{\text{k}}_{1}\text{sig}{\left(\epsilon_{\text{G}}\right)}^{{\text{g}}_{1}}-2{\text{k}}_{2}\epsilon_{\text{G}}\epsilon_{\text{G}}+2\epsilon_{\text{G}}w_{1}+2\epsilon_{\text{G}}\epsilon_{\text{X}}$$

The analysis in the previous section establishes that the error $\epsilon_{\text{X}}$ converges to the set ${\text{D}}_{\text{X}}$ within a finite time ${\text{T}}_{\text{X}}$. Consequently, for all $t\geq {\text{T}}_{\text{X}}$, we have $|\epsilon_{\text{X}}|\leq \Delta_{\text{X}}$. Let ${\bar{w}}_{1}$ denote the upper bound of the disturbance term $w_{1}$. Based on Equation 19, we derive the following inequality.

${\dot{V}}_{\text{G}}\leq -2{\text{k}}_{1}|\epsilon_{\text{G}}{|}^{{1+g}_{1}}-2{\text{k}}_{2}\epsilon_{\text{G}}^{2}+2|\epsilon_{\text{G}}|({\bar{w}}_{1}+\Delta_{\text{X}})$Introducing arbitrary constants ${\text{θ}}_{\text{G}1},{\text{θ}}_{G2}\in (0,1)$, we can decompose the Lyapunov derivative as.

(20) $$\begin{array}{l} {\dot{V}}_{\text{G}}\leq -2{\text{k}}_{1}(1-{\text{θ}}_{\text{G}1})V_{\text{G}}^{{\text{(1+g}}_{1})/2}-2{\text{k}}_{2}(1-{\text{θ}}_{G2})V_{\text{G}}-\\ \;2{\text{k}}_{1}{\text{θ}}_{\text{G}1}|\epsilon_{\text{G}}{|}^{{1+g}_{1}}-2{\text{k}}_{2}{\text{θ}}_{G2}\epsilon_{\text{G}}^{2}+2|\epsilon_{\text{G}}|({\bar{\text{w}}}_{1}+{\text{Δ}}_{\text{X}}) \end{array}$$

Define the auxiliary sets and their associated thresholds:


$$\begin{array}{l} {\text{D}}_{\text{G}1}≜\{\epsilon_{\text{G}}:|\epsilon_{\text{G}}|<{\text{Δ}}_{\text{G}1}\},\;{\text{Δ}}_{\text{G}1}≜{\text{k}}_{1}^{-1}{\text{θ}}_{\text{G}1}^{-1}{(}^{{\bar{w}}_{1}}{+}^{{\text{Δ}}_{\text{X}}}{)}^{1/{\text{g}}_{1}}\\ {\text{D}}_{\text{G}2}≜\{\epsilon_{\text{G}}:|\epsilon_{\text{G}}|<{\text{Δ}}_{\text{G}2}\},\;{\text{Δ}}_{\text{G}2}≜{\text{k}}_{2}^{-1}{\text{θ}}_{\text{G}2}^{-1}({\bar{w}}_{1}+{\text{Δ}}_{\text{X}}) \end{array}$$

$${\text{D}}_{\text{G}}≜{\text{D}}_{\text{G}1}\cap {\text{D}}_{\text{G}2}$$

When $\epsilon_{\text{G}}$ is outside the set ${\text{D}}_{\text{G}}$, the last three terms on the right side of inequality (20) satisfy the following inequality.


$$-2{\text{k}}_{1}{\text{θ}}_{\text{G}1}|\epsilon_{\text{G}}{|}^{{1+g}_{1}}-2{\text{k}}_{2}{\text{θ}}_{G2}\epsilon_{\text{G}}^{2}+2|\epsilon_{\text{G}}|({\bar{\text{w}}}_{1}+{\text{Δ}}_{\text{X}})\leq 0$$

Substituting the above equation into Equation 20 yields.

(21) $${\dot{V}}_{\text{G}}\leq -2{\text{k}}_{1}(1-{\text{θ}}_{\text{G}1})V_{\text{G}}^{{\text{(1+g}}_{1})/2}-2{\text{k}}_{2}(1-{\text{θ}}_{G2})V_{\text{G}}$$

Since inequality (21) always holds when $\epsilon_{\text{G}}$ is outside set ${\text{D}}_{\text{G}}$, this indicates that $\epsilon_{\text{G}}$ will converge and stabilize within the set $|\epsilon_{\text{G}}|<{\text{Δ}}_{\text{G}}$, where ${\text{Δ}}_{\text{G}}≜\min$ $\left\{{\text{Δ}}_{\text{G}1},{\text{Δ}}_{G2}\right\}$. Define ${\text{T}}_{\text{G}}$ as the time required for $\epsilon_{\text{G}}$ to start from the moment it enters t=0 until it finally converges to the set ${\text{D}}_{\text{G}}$. Below, we calculate an upper bound for the convergence time of ${\text{T}}_{\text{G}}$. Note that both terms on the right side of Equation 21 accelerate the system’s convergence, so must be less than the convergence time corresponding to each term acting individually on the system. Next, we calculate the convergence times corresponding to each of these two terms acting individually on the system. Equation 21 satisfies the following inequality.

(22) $${\dot{V}}_{\text{G}}\leq -2{\text{k}}_{1}(1-{\text{θ}}_{\text{G}1})V_{\text{G}}^{{\text{(1+g}}_{1})/2}$$

According to Lemma 2, and noting that $V(x({\text{T}}_{\text{Δ}}))$ in Lemma 2 corresponds to $V_{\text{G}}(T_{G1})=\Delta_{\text{G}}^{2}$ here, and $V(x_{0})$ corresponds to $\epsilon_{\text{G}}{\left({\text{T}}_{\text{X}}\right)}^{2}$, we can directly obtain the upper bound of the convergence time ${\text{T}}_{\text{G}1}$ corresponding to inequality (Equation 22) as.

(23) $${\text{T}}_{\text{G}1}\leq {\text{T}}_{\text{X}}+({\text{Δ}}_{\text{G}}^{1-{\text{g}}_{1}}-\epsilon_{\text{G}}{\left({\text{T}}_{\text{X}}\right)}^{2}){\left[{\text{k}}_{1}(1-{\text{θ}}_{\text{G}1})({\text{g}}_{1}-1)\right]}^{-1}$$

Equation 21 also satisfies the following inequality.

(24) $${\dot{V}}_{\text{G}}\leq -2{\text{k}}_{2}(1-{\text{θ}}_{G2})V_{\text{G}}$$

The above equation is highly similar to inequality (14) from the previous subsection. The analysis will not be repeated here; please refer to the analysis process from Equations 14–17 to obtain the upper bound ${\text{T}}_{G2}$ on the convergence time corresponding to inequality (Equation 24).

(25) TG2≤TX+k2 (−11−θG2)−1(lnϵG(TX)−lnΔG)

Based on the two calculated convergence times, (Equations 23) and (Equation 25), it can be concluded that the time ${\text{T}}_{\text{G}}$ required for blood glucose error $\epsilon_{\text{G}}$ to converge to $|\epsilon_{\text{G}}|<{\text{Δ}}_{\text{G}}$ must satisfy condition ${\text{T}}_{\text{G}}\leq \min\{{\text{T}}_{\text{G}1},{\text{T}}_{G2}\}$. The theoretical analysis is complete.

The above theoretical derivation process can be summarized as the following theorem:

Theorem: Consider the blood glucose control model (1) for T1DM patients affected by disturbances. If the insulin infusion rate based on backstepping control and power exponent parameters is designed as in Equations 2–4, then the artificial pancreas can regulate the patient’s blood glucose to near the desired blood glucose level ${\text{G}}_{\text{d}}$ in a finite time ${\text{T}}_{\text{G}}$, with a steady-state accuracy of $|\epsilon_{\text{G}}|<{\text{Δ}}_{\text{G}}$.

Proof: The stability is proven via a three-step backstepping analysis. First, the insulin tracking error $\epsilon_{\text{I}}$ is shown to converge in a finite time ${\text{T}}_{\text{I}}$ time to a set ${\text{D}}_{\text{I}}$. Then, based on this boundedness, the remote insulin error $\epsilon_{\text{X}}$ is proven to converge in a finite time ${\text{T}}_{\text{X}}$ to a set ${\text{D}}_{\text{X}}$. Finally, given the bounded $\epsilon_{\text{X}}$, the glucose error $\epsilon_{\text{G}}$ is driven to its ultimate bound ${\text{Δ}}_{\text{G}}$ in a finite time ${\text{T}}_{\text{G}}$. The detailed mathematical expression for the aforementioned finite times ${\text{T}}_{\text{I}}$, ${\text{T}}_{\text{X}}$, ${\text{T}}_{\text{G}}$ and convergence sets ${\text{D}}_{\text{I}}$, ${\text{D}}_{\text{X}}$, ${\text{Δ}}_{\text{G}}$ are given in the stability analysis, as detailed in the previous derivation.

*Remark 2*: The lemmas used in the above stability analysis are provided here. Consider the following system:

(26) $$\dot{\text{x}}(t)=\text{f}(\text{x}(t)),\;\text{f}(0)=0,\;t_{0}=0,\;{\text{x}}_{0}≜\text{x}(0)$$

In the formula $\text{x}(t)\in {\text{R}}^{n}$, $\text{f}:\text{U}\to {\text{R}}^{\text{n}}$ are functions from the domain U containing the origin to an n -dimensional space ${\text{R}}^{\text{n}}$, where $0\in {\text{R}}^{\text{n}}$ represents the zero vector and ${\text{x}}_{0}$ represents the initial state. Based on the system above, the following lemmas on finite-time and sub-fixed-time stability are introduced.

Lemma 1 (Finite-Time Stability) (32): For system (Equation 26), if there exists a first-order differentiable positive definite function V(x) such that.

(27) $$\dot{V}(\text{x})\leq -{\text{b}}_{1}V{\left(\text{x}\right)}^{\text{p}}$$

where ${\text{b}}_{1}$ and p are positive constants satisfying condition p∈(0,1), the system (Equation 26) is finite-time stable, and the convergence time $\text{T}(x_{0})$ satisfies.

(28) $$\text{T}(x_{0})\leq {\text{b}}_{1}^{-1}{\left(1-\text{p}\right)}^{-1}V{\left({\text{x}}_{0}\right)}^{1-\text{p}}$$

Lemma 2 (Sub-Fixed-Time Stability) (33) For system (Equation 26), if there exists a first-order differentiable positive definite function V(x) such that.

(29) $$\dot{V}(\text{x})\leq -{\text{b}}_{2}V{\left(\text{x}\right)}^{\text{g}}$$

where ${\text{b}}_{2}$and g are positive constants satisfying condition g>1, the system (Equation 26) is sub-fixed-time stable. This means that if the steady-state accuracy of the system state is defined as $\text{Δ}≜V(\text{x}({\text{T}}_{\text{Δ}}))$, and ${\text{T}}_{\text{Δ}}$ represents the time required for the system state x to converge to Δ, then the convergence time ${\text{T}}_{\Delta }$ satisfies.

(30) $${\text{T}}_{\text{Δ}}\leq ({\text{Δ}}^{1-\text{g}}-V({\text{x}}_{0})){\left[{\text{b}}_{2}(\text{g}-1)\right]}^{-1}$$

Remark 3. The insulin infusion rate designed in this paper does not incorporate a saturation constraint on the control input. However, in practical applications, the physical limitations of the insulin pump must be considered. The actual infusion rate must satisfy $0<sat(u)<u_{\max}$, where $u_{\max}$ is the pump’s maximum delivery rate. Notably, following meal consumption—especially with high carbohydrate intake—a significant rise in blood glucose concentration often causes the controller to demand a high insulin infusion rate. This frequently triggers saturated infusion from the pump. This phenomenon does not indicate control failure; rather, it demonstrates that the control algorithm is fully utilizing the pump’s delivery capacity to infuse insulin at the maximum rate, functioning similarly to a pre-meal bolus supplement in open-loop therapy. Since the duration of sustained saturated infusion is not accounted for in the stability analysis presented in this paper, the actual time required for blood glucose to reach the target range will be longer than the theoretical prediction. Considering that the insulin pump does not normally remain in a saturated state indefinitely, the advantages of the proposed power exponent control—such as its finite-time convergence property—may be affected but not entirely invalidated by control saturation. It should be clarified that closed-loop stability analysis under control input saturation is not the primary focus of this research, and related findings in the field of artificial pancreas systems remain relatively limited to date. Nonetheless, designing insulin infusion strategies with saturation constraints and analyzing their closed-loop performance constitute a research direction of significant theoretical and practical value and are anticipated to become focal points of future research in this field.

## 3 Simulation

This section presents three simulation cases designed to compare the glucose-lowering effectiveness of the proposed controller (2) with that of the controller in Ref (27). In the first simulation case, the control parameters are adjusted so that the glucose-lowering curves and insulin usage of both control schemes achieve essentially identical outcomes. The purpose of this set of simulations is to establish a unified performance benchmark for a fair comparison in subsequent simulations. This approach ensures that any performance differences observed in the more challenging simulations that follow can be unequivocally attributed to the inherent capabilities of the algorithms in handling difficult scenarios, rather than to suboptimal parameter tuning. Under the premise of keeping the control parameters unchanged, the second and third simulations progressively incorporate the effects of high initial blood glucose and daily meals. These simulations test the control schemes’ ability to regulate isolated acute hyperglycemia and to respond to more complex and challenging scenarios.

The model parameter values used in the simulations are provided below. The parameters for the patient in the blood glucose model (1) are taken from the U.S. Food and Drug Administration (FDA)-accredited diabetes simulation software (31, 34), the UVA/Padova T1DM simulator. Using a parameter identification method, Reference (31) extracted and calculated the average parameters of the BMM from the data of 11 adult patients in this software, as detailed in **Table 1**.

**Table 1:** Patient parameters in glucose model (1) (31).

Parameter	Unit	Value
c_1_	min^-1^	0.0023
c_2_	min^-1^	0.0118
c_3_	L mU min^-2^	7.2×10^-7^
c_4_	min^-1^	0.009
c_5_	L^-1^	0.009
G_b_	mg/dL	119
I_b_	mU/L	15.2

Based on clinical practice, the target blood glucose level (i.e., the control objective) is set to ${\text{G}}_{\text{d}}=108$ mg/dL, and the initial values of X(t) and I(t) are set to X(0)=0 min^-1^ and I(0)=15 mU/L. The mathematical expressions of disturbances $w_{11}$, $w_{12}$ and $w_{21}$ in the blood glucose model (1) are as follows.


$${\dot{w}}_{11}=-{\text{c}}_{6}w_{11}$$

$$w_{12}=0.1\sin(0.1t+1)+0.1\cos(0.05t+2)\text{ mg/dl/}\min$$

$$w_{21}=0.1\sin(0.1t)+20r(t)\text{ mU/L/}\min$$

where, the value of ${\text{c}}_{6}$ are taken from Ref (35). r(t) is a Gaussian random variable with a mean of 0, a variance of 1×10^-4^, and a sampling interval of 10 minutes. Its role is to introduce a certain degree of randomness to the disturbance to better reflecting real-world conditions. According to Ref (19), the saturation function sat (·) in *w*_22_ is configured with an upper safety limit of 50 mU/min and a lower limit of 0.

The sub-fixed-time controller designed in this paper belongs to the category of power exponent control. Research on power exponent control within the field of artificial pancreas control is extremely limited. Currently, it appears that only the homogeneous control proposed in Ref (27), which also focuses on artificial pancreas control, shares this classification with the method presented here. To ensure a rational and fair comparison, we have selected the method from Ref (27) as the benchmark. A systematic comparison between our method and this benchmark will be conducted under identical simulation scenarios to evaluate their performance on key metrics such as convergence speed and robustness. The specific structure of the homogeneous controller in Ref (27) is provided below.

(31) $$u={\text{c}}_{3}^{-1}{\text{c}}_{5}^{-1}[-{\text{k}}_{2}\text{sig}{\left(e_{1}\right)}^{{\text{p}}_{1}}-{\text{k}}_{3}\text{sig}{\left(e_{2}\right)}^{2{\text{p}}_{1}/(1+{\text{p}}_{1})}-f(t)]$$

$$e_{1}=X-X_{d},\;e_{2}=\dot{X}-{\dot{X}}_{d},\;e_{G}=G(t)-{\text{G}}_{\text{d}}$$

$$X_{d}=-{\text{c}}_{1}G^{-1}(G-G_{b})+{\text{k}}_{1}G^{-1}e_{G}$$

$$f(t)=\varphi m+{\text{c}}_{2}^{2}X-{\text{c}}_{3}({\text{c}}_{2}+{\text{c}}_{4})I-hn^{2}$$

$$\varphi =G^{-1}[{\text{k}}_{1}-{\text{c}}_{1}G^{-1}{\text{G}}_{\text{b}}-{\text{k}}_{1}G^{-1}e_{G}]$$

$$m=({\text{c}}_{1}+X)({\text{c}}_{1}G-{\text{c}}_{1}G_{b}+GX)+{\text{c}}_{2}GX-{\text{c}}_{3}GI$$

$$n=-{\text{c}}_{1}(G-{\text{G}}_{\text{b}})-GX$$

$$h=2G^{-2}({\text{c}}_{1}G^{-1}{\text{G}}_{\text{b}}+{\text{k}}_{1}G^{-1}e_{G}-{\text{k}}_{1})$$

The model parameters $c_{i}$ (where i = 1, …, 5) are the glucose model parameters, as defined in this paper. The controller parameters include the gain parameters k_1_, k_2_, k_3_, and the power exponent parameter p_1_. Following the setup in Ref (27), their values are set as: k_1_ = 0.005, k_2_ = 0.001, k_3_ = 0.03, p_1_ = 0.6.

The simulation results of the three sets are as follows.

### 3.1 Simulation 1: Baseline scenario with standard meals and parameter tuning

The objective of this simulation is to establish a benchmark for comparison in subsequent simulations, rather than to directly evaluate the relative merits of the two methods. Specifically, within this benchmark scenario, we adjust the parameters of the proposed controller to align the output responses of both schemes. This ensures that the glucose-lowering curves and insulin usage are essentially identical. Consequently, any performance differences observed in subsequent simulations can be conclusively attributed to the algorithms’ inherent capabilities in handling complex scenarios, rather than to incidental effects of initial parameter settings.

First, the mealtimes for the simulated patient and the initial rates of blood glucose change after each meal are set. The patient is assumed to consume meals daily at 6:00, 12:00, and 18:00. The initial rates of meal-induced blood glucose fluctuation at these times are *w*_11_(6:00)=2.5 mg/dL/min, *w*_11_(12:00)=5 mg/dL/min, and *w*_11_(18:00)= 2.5 mg/dL/min, respectively. The initial blood glucose concentration is set at *G*(6:00)=108 mg/dL.

The control parameters are adjusted to align the output response of the proposed controller with that of the homogeneous controller in Ref (27) ensuring that their glucose-lowering curves and insulin infusion amounts are essentially identical. After adjustment, the parameters for the proposed controller are determined as follows: k_1_=k_2_ = 0.1, k_3_ = 0.15, k_4_ = 3.75, g_1_ = 1.2, and p_1_ = 0.8. The parameter tuning procedure can be summarized in four steps. First, following the general design principles of power exponent control, the power exponent parameters are set to g_1_ = 1.2 and p_1_ = 0.8. Second, since the terms associated with the gain parameters k_1_ and k_2_ share the same order of magnitude and have similar effects, we set k_1_=k_2_. Third, a trial-and-error method is employed to adjust the gain parameters until the glucose-lowering curve achieves preliminary stability. Finally, based on the following empirical insights gained during our tuning process, the parameters are fine-tuned: the values of k1 and k_3_ are negatively correlated with both the speed of glucose reduction and the insulin dose, whereas the value of k_4_ is positively correlated with both; simultaneously, k3 and k4 jointly influence the rising slope of the insulin dose curve. By iteratively adjusting the gain parameters according to the above rules, the glucose-lowering and insulin dose curves of the proposed controller are ultimately brought into near coincidence with the corresponding curves of the homogeneous controller in Ref (27).

**Figure 2:**
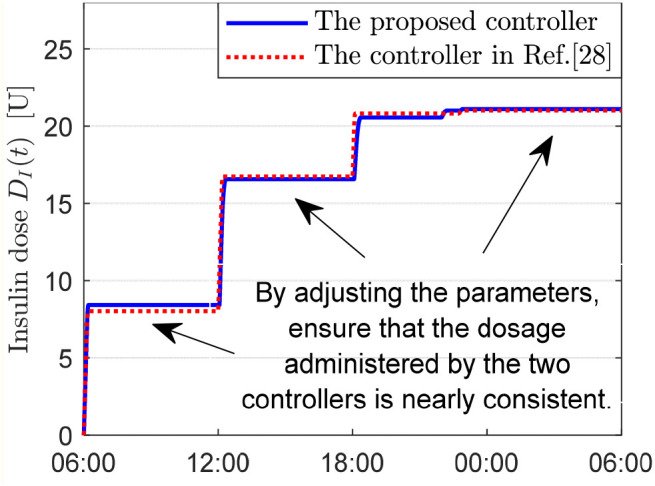
Insulin dose for scenario 1.

**Figure 3:**
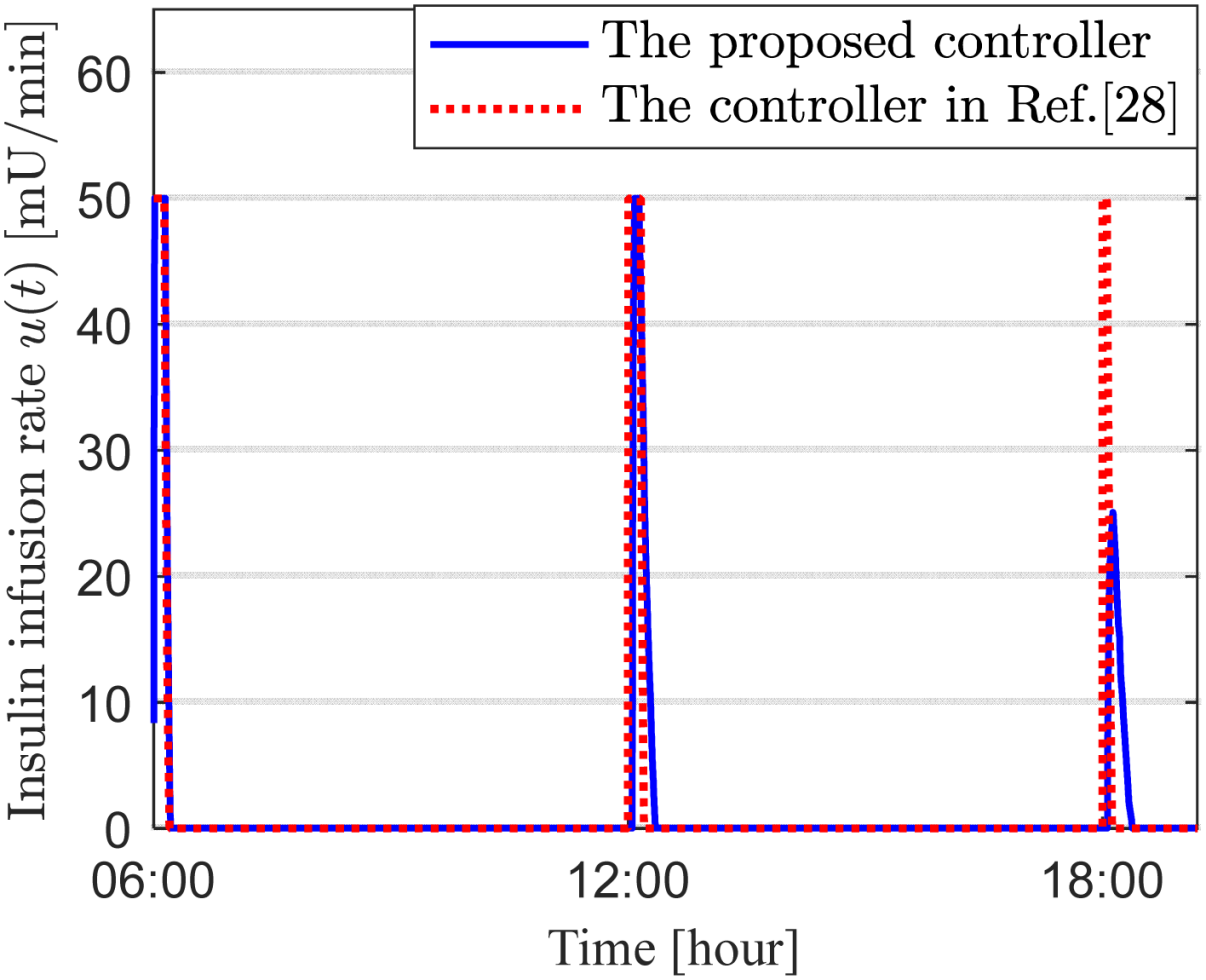
Insulin infusion rate for scenario 1.

**Figure 4:**
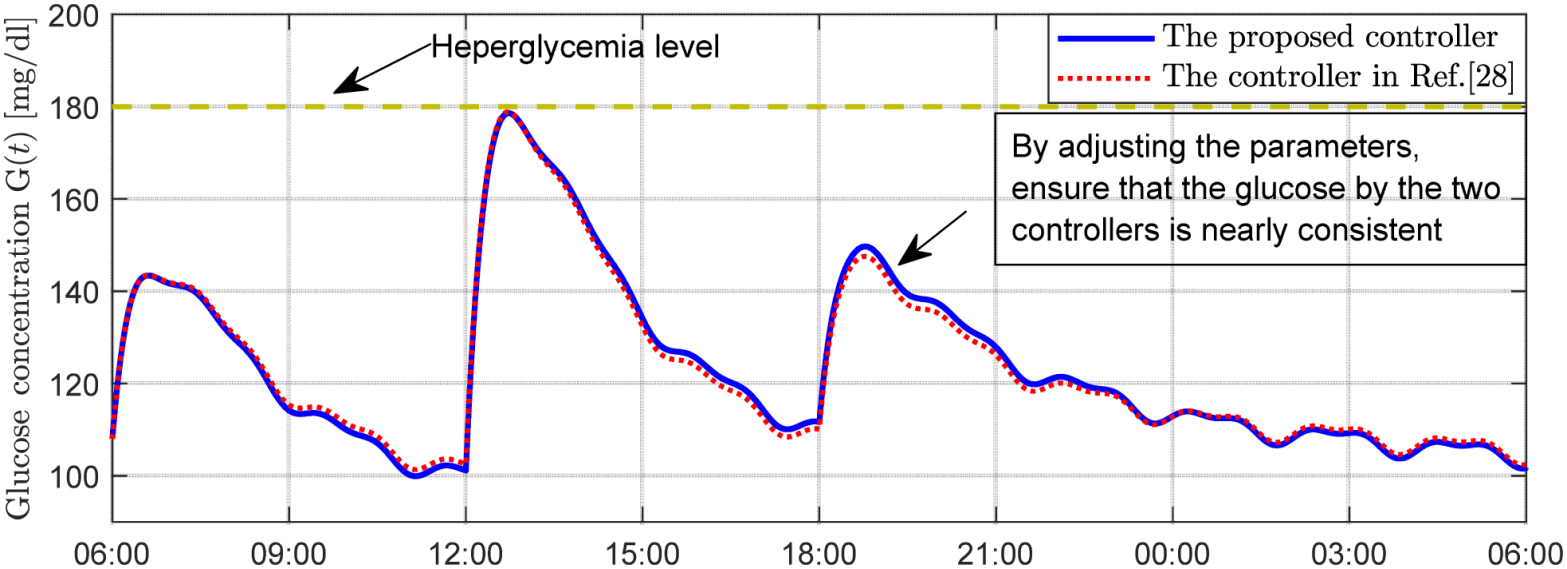
Plasma glucose concentration for scenario 1.

Under the two control schemes, the curves representing insulin usage, infusion rate, and full-day blood glucose concentration are shown in **Figures 2**-**4**, respectively. **Figure 2** compares the cumulative insulin doses of the two control schemes, expressed by the formula $D_{I}=0.001$ × ∫u(t) dt [U]. **Figure 3** presents the corresponding insulin infusion rates. **Figure 4** illustrates the temporal variation of the patient’s blood glucose concentration throughout the day. As observed in **Figures 2**, **3**, the glucose-lowering curves and insulin dose curves for both control schemes are essentially consistent.

### 3.1 Simulation 2: Acute hyperglycemia without meals

**Figure 5:**
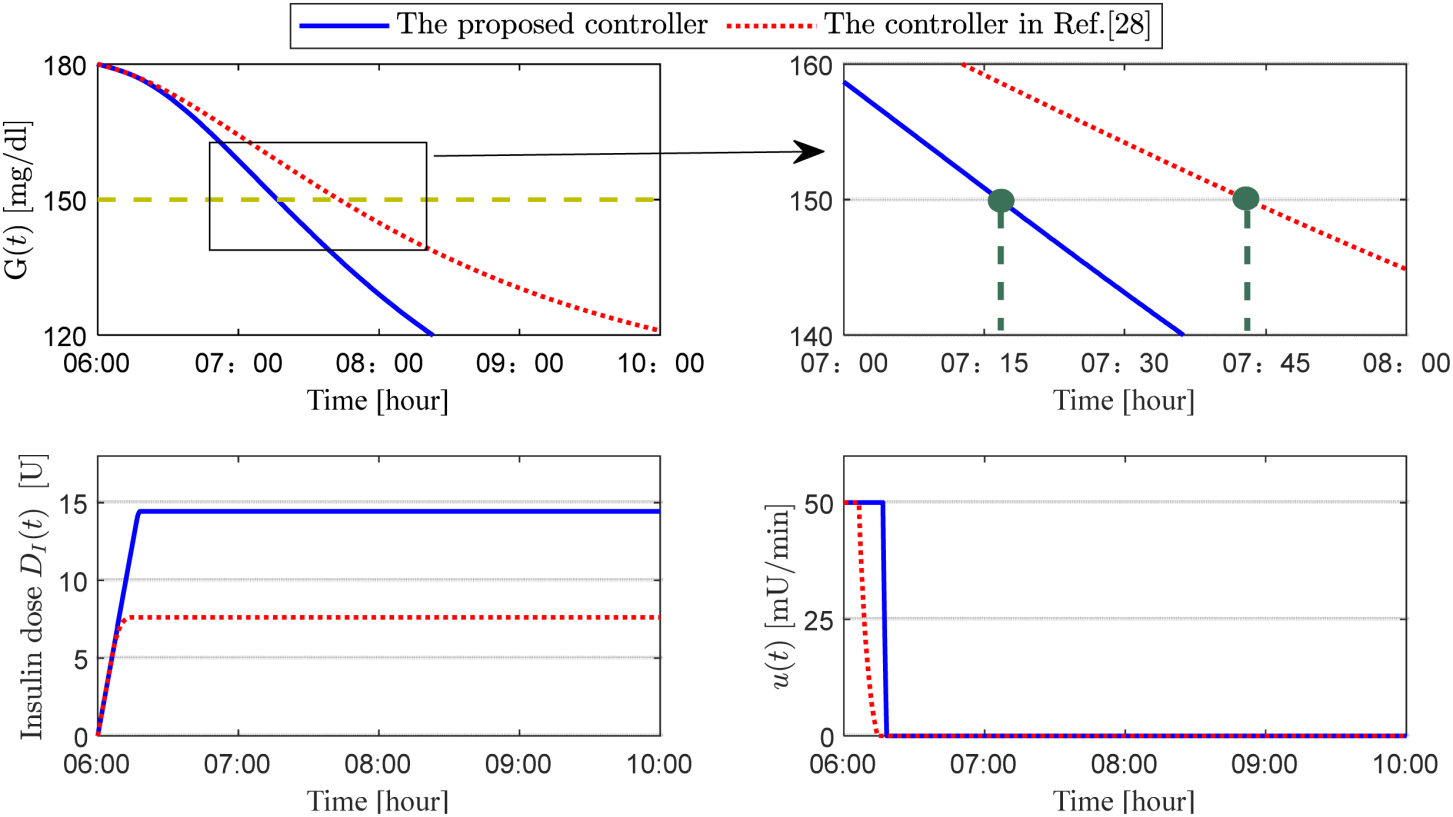
Responses of plasma glucose concentration, insulin dose and insulin infusion rate for scenario 2.

This simulation assesses the effectiveness of two control methods in lowering blood glucose levels in hyperglycemic patients over a brief timeframe. Meal consumption is excluded from the analysis. It is assumed that the patient’s blood glucose concentration at 6:00 AM is G(6:00)=180 mg/dL, and the goal is to decrease it to under 150 mg/dL within two hours. **Figure 5** illustrates the patient’s blood glucose concentration, insulin dosage, and insulin infusion rate. A detailed analysis of these simulation results is provided in the following section, the Discussion.

### 3.3 Simulation 3: Composite scenario (hyperglycemia & meals)

**Figure 6:**
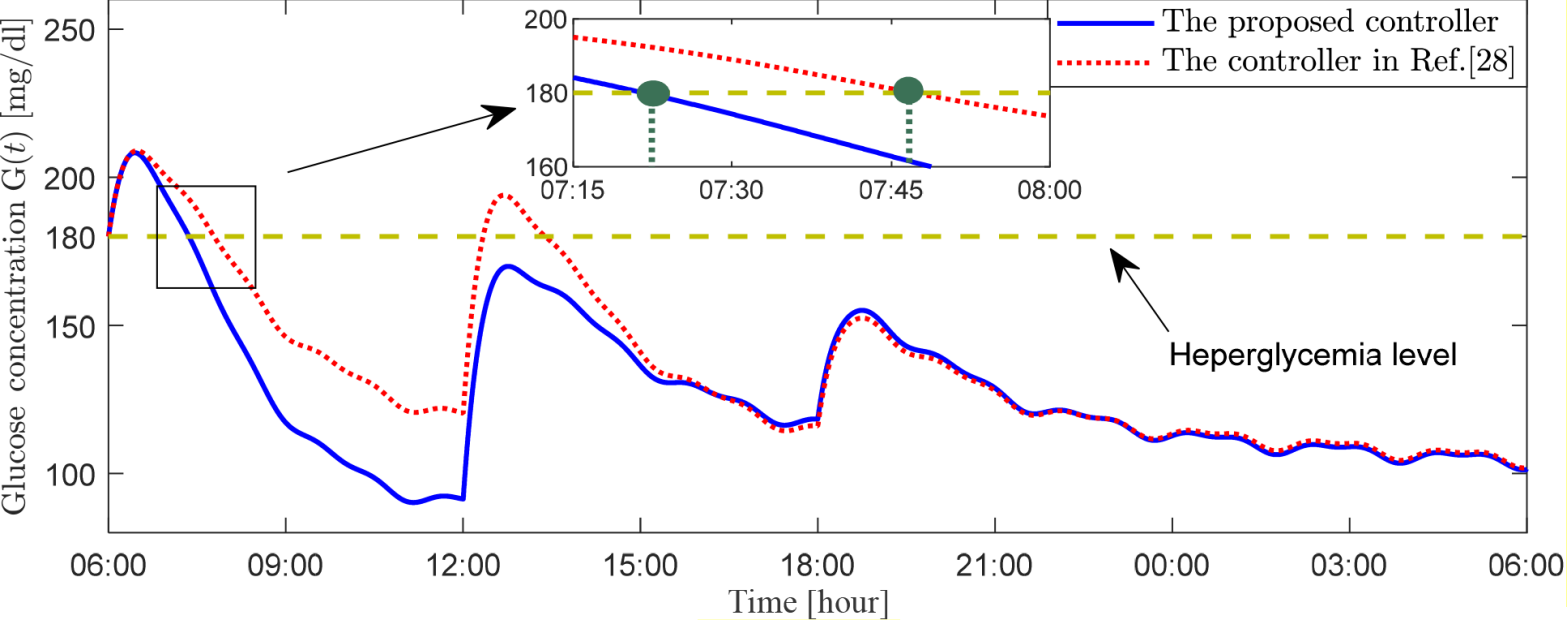
Plasma glucose concentration for scenario 3.

**Figure 7:**
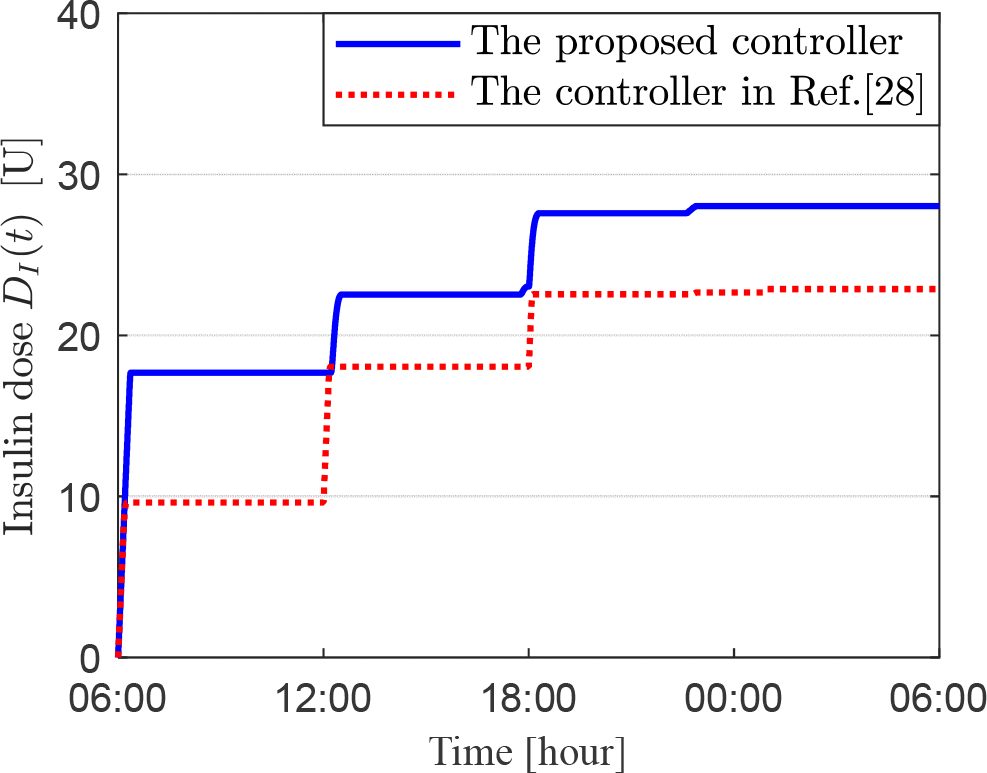
Insulin dose for scenario 3.

**Figure 8:**
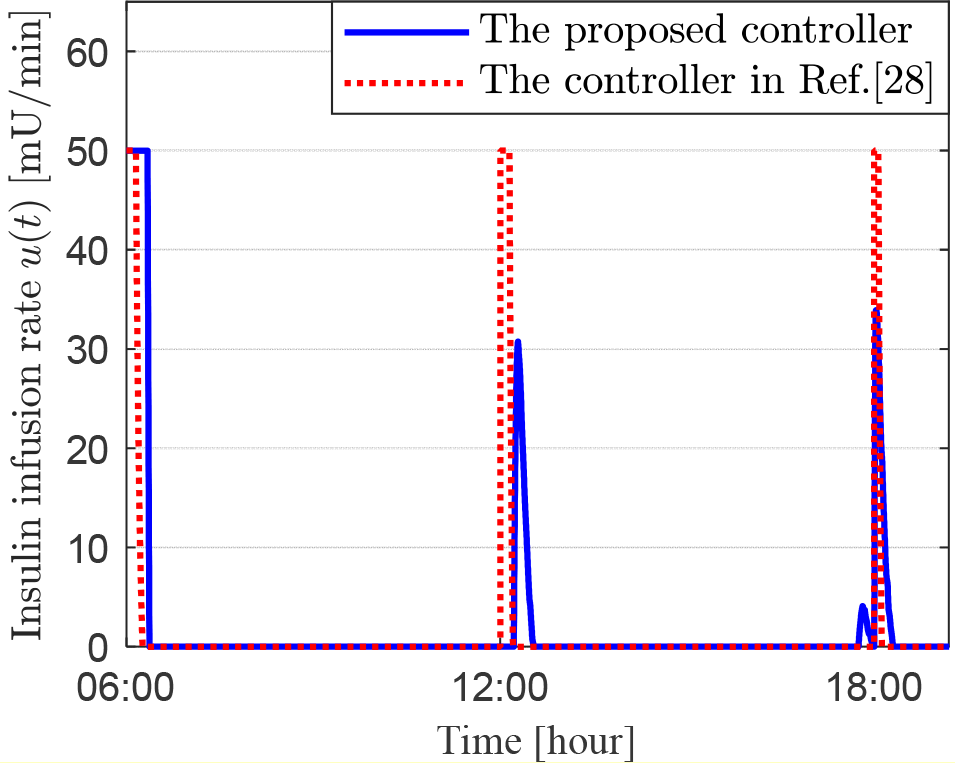
Insulin infusion rate for scenario 3.

This simulation combines the two previously described simulations to assess the effectiveness of two control strategies in lowering blood glucose levels while accounting for three daily meals and an initial elevated blood glucose level of 180 mg/dL. **Figures 6**–**8** display the patient’s blood glucose concentration, insulin dosage and insulin infusion rate over time throughout the day, respectively. A detailed analysis of these simulation results is provided in the following section, the Discussion.

## 4 Discussion

In the first set of simulations, the controller parameters were adjusted so that the glucose-lowering effects of the two control schemes were consistent, and both met the clinical standards for an ideal artificial pancreas under daily conditions. The purpose of tuning the control performance of both schemes to an identical level was to establish a unified performance benchmark for a fair comparison in the subsequent two simulation sets. As shown in **Figures 2**-**4**, the insulin usage and 24-hour blood glucose fluctuation curves for the two control schemes are nearly identical. The latest diabetes treatment guidelines (36, 37) recommend that the glycemic targets for patients with type 1 diabetes are a preprandial blood glucose of 80–130 mg/dL and a postprandial blood glucose (1–2 hours after meals) of less than 180 mg/dL. As shown in **Figure 4**, the 24-hour blood glucose levels under both controllers achieve these target ranges, with blood glucose dynamically fluctuating between 80 and 180 mg/dL throughout the day. Specifically, preprandial blood glucose levels remain within 90–120 mg/dL, and 2-hour postprandial blood glucose levels remain within 120–160 mg/dL. This glycemic control range is ideal, with no occurrences of hypoglycemia or peak hyperglycemia. These results indicate that the two controllers perform comparably throughout the day when their parameters are aligned and fasting blood glucose is appropriate, suggesting they are comparable for subsequent comparisons under altered initial conditions.

In the second set of simulations, we considered the scenario in which a patient begins using the artificial pancreas only after experiencing elevated blood glucose levels. Consequently, the initial blood glucose value was increased from 108 mg/dL to 180 mg/dL. Simultaneously, meal disturbances were removed from the closed-loop glucose system to specifically evaluate the algorithm’s pure regulatory capability in managing isolated acute hyperglycemia. As shown in **Figure 5**, the proposed controller (2) reduced blood glucose to 150 mg/dL by 07:15, whereas the controller in Ref (27) did not achieve this reduction until 07:45. Thus, the proposed controller reached this target 30 minutes earlier (see the magnified blood glucose plot in the upper-right corner of **Figure 5**). From a clinical safety perspective, an excessively rapid glucose-lowering strategy is inadvisable, as it can cause patient intolerance and significantly increase the risk of hypoglycemia and cardiovascular events. Clinical symptoms may include dizziness, blurred vision, or even loss of consciousness, which can be life-threatening in severe cases. Therefore, clinical guidelines generally recommend that the glucose-lowering rate for significant hyperglycemia be controlled within 50–100 mg/dL per hour, while for routine blood glucose regulation, the recommended rate is 10–30 mg/dL per hour. The glucose-lowering rate of the proposed controller (2) is approximately 24 mg/dL per hour, which clearly aligns with these clinical recommendations. This indicates that the proposed controller (2) can regulate blood glucose more rapidly and flexibly while ensuring patient safety. According to the data in **Figure 5**, compared to the controller in Ref (27), the proposed controller (2) utilizes the insulin pump’s infusion capacity more effectively. Specifically, when starting from a hyperglycemic state, the proposed controller (2) maintains a longer period of saturated infusion based on actual conditions (see the insulin infusion rate plot in the lower-right corner of **Figure 5**), thereby delivering a higher cumulative insulin dose (see the insulin dose plot in the lower-left corner of **Figure 5**). This increases the insulin concentration in the patient’s body and, while maintaining safety, ultimately shortens the time required to lower blood glucose, achieving the goal of effectively preventing hyperglycemia.

In the third set of simulations, we constructed a composite scenario that more closely approximates clinical complexity. This scenario retains the high initial blood glucose level from the second simulation while incorporating the three-meal daily glucose disturbances from the first simulation. This design enables a comprehensive evaluation of the two control methods’ ability to maintain all-day glucose stability under sustained and dynamic disturbances. Postprandial blood glucose rises due to carbohydrate absorption, triggering the artificial pancreas’s feedback control to infuse insulin and lower glucose levels, resulting in a typical postprandial glucose peak. The key to evaluating the performance of a glucose control algorithm lies in its effectiveness in suppressing the magnitude of this peak fluctuation and accelerating the rate at which glucose declines from the peak back to a safe threshold, typically considered to be below 180 mg/dL. **Figure 6** presents the all-day blood glucose fluctuation curves for the two controllers. Taking the morning period in **Figure 6** as an example, the blood glucose under both schemes rose to a peak of 209 mg/dL at 06:55. After this peak, the blood glucose under the proposed controller (2) decreased to 180 mg/dL by 07:22, whereas the control scheme in Ref (27) achieved this level at 07:47. The former was 25 minutes faster. Furthermore, the proposed controller (2) lowered blood glucose to 92 mg/dL before lunch, providing favorable conditions for maintaining glucose within an ideal range after the lunch meal. In contrast, the controller in Ref (27) reduced pre-lunch glucose to 120 mg/dL. Although this meets the guideline-recommended range of 80–130 mg/dL, its higher baseline level led to less ideal post-lunch glucose control, with values even temporarily exceeding the target control limit of 180 mg/dL, representing slightly inferior performance. Although the control scheme in Ref (27) maintained blood glucose within the target range for over 70% of the time, compared to the proposed controller (2), the latter demonstrates significant advantages in terms of time-in-range, speed of glucose regulation, and regulatory flexibility. As observed from **Figures 6**-**8**, under the composite challenge of high initial glucose combined with periodic meal disturbances in the third simulation, the proposed controller (2), by maintaining a longer duration of insulin saturation infusion (**Figure 8**), delivered a higher insulin dose during critical periods (**Figure 7**). Consequently, the closed-loop system attained a faster initial glucose-lowering rate and stronger postprandial disturbance rejection capability, significantly optimizing the overall control performance of the all-day blood glucose profile.

Based on the integrated discussion of the results from the three simulation sets, it can be concluded that although both controllers belong to the category of non-smooth control methods incorporating power exponent parameters, the proposed controller (2) exhibits a faster glucose-lowering effect when the system’s initial state is far from the equilibrium point, as demonstrated in the case of initial hyperglycemia in the second simulation. Furthermore, when the system is subjected to significant disturbances—such as meal disturbances and those arising from control saturation considered in the third simulation—the proposed controller (2) demonstrates stronger disturbance rejection capabilities. The primary reason for these advantages of the proposed controller (2) over the controller in Ref (27) lies in its design, which incorporates an additional power exponent parameter, g1, and achieves sub-fixed-time stability. This conclusion aligns with that of the seminal Ref (27), which first introduced the concept of sub-fixed-time stability. Therefore, incorporating dual power exponent parameters—one less than 1 and one greater than 1—into the artificial pancreas controller can further enhance the glucose-lowering efficacy of the control system. This enhancement is concretely manifested as improved disturbance rejection capability of the closed-loop glucose system and superior robustness to variations in the patient’s initial blood glucose level.

## 5 Conclusion

Blood glucose levels in diabetic patients can fluctuate significantly after meals, and factors such as the secretion of hyperglycemic hormones and stress conditions can also cause blood glucose fluctuations. Therefore, studying the anti-interference ability of artificial pancreas systems against blood glucose fluctuations is an important aspect of improving their blood glucose-lowering effectiveness. This paper focuses on the control system of the artificial pancreas, primarily addressing the issues that existing related control technologies are largely lack system stability analysis. Specifically considering the impact of patient meal disturbances and insulin infusion process disturbances on blood glucose fluctuations. A new insulin infusion rate scheme with two power exponent parameters is proposed based on backstepping control theory. Using Lyapunov stability principles, a rigorous stability analysis of the closed-loop system is conducted, and complete mathematical expressions for the time required to lower blood glucose and the error accuracy are provided. This offers strong mathematical theoretical support for achieving closed-loop blood glucose regulation in artificial pancreas systems. Three simulation experiments demonstrate that, when managing blood glucose variations resulting from uncertainties in daily meals and infusion processes, the proposed control method—enhanced by incorporating a power exponent parameter—achieves a quicker reduction in glucose levels compared to the homogeneous control. Moreover, the blood glucose peaks throughout the day are consistently lower, indicating a superior capability to mitigate blood glucose fluctuations in patients.

## Data Availability

The original contributions presented in the study are included in the article/supplementary material. Further inquiries can be directed to the corresponding author/s.

## Appendix

**Table:** Summary of Variables and Parameters.

Symbol	Description
*G*	Blood glucose concentration
*X*	Insulin’s glucose-lowering effect
*I*	Insulin concentration
*u*	Insulin infusion rate
G_b,_ I_b_	Basal glucose and insulin concentration
c_1_, c_2_, c_3_, c_4_, c_5_	Physiological parameters in BMM
*w*_1_, *w*_11_, *w*_12_	Glucose dynamics disturbances
*w*_2_, *w*_21_, *w*_22_	Insulin dynamics disturbances
$I_{\text{d}}$	Virtual control law for *I*-subsystem
$X_{\text{d}}$	Virtual control law for *X*-subsystem
$\epsilon_{\text{G}}≜G-$ ${\text{G}}_{\text{d}}$	Blood glucose error
$\epsilon_{\text{X}}≜X-X_{\text{d}}$	Remote insulin error
$\epsilon_{\text{I}}≜I-I_{\text{d}}$	Insulin tracking error
G_d_	Target blood glucose concentration
*m*, *m*_1_, *m*_2_, *m*_3_, *m*_4_, *m*_5_, *l*	Auxiliary variables in controller
k_1_, k_2_, k_3_, k_4_	Gain parameters in controller
g_1_, p_1_	Power exponent parameters in controller
$\theta_{\text{I}}$, $\theta_{\text{X}}$, $\theta_{G1}$, $\theta_{G2}$	Stability Analysis Parameters
T_I_, T_X_, T_G_	Convergence time of $\epsilon_{\text{I}}$, $\epsilon_{\text{X}}$ and $\epsilon_{\text{G}}$
T_G1_, T_G2_	Sub-time of T_G_
D_I_, D_X_, D_G_	Ultimate bounded sets for $\epsilon_{\text{I}}$, $\epsilon_{\text{X}}$ and $\epsilon_{\text{G}}$
$\Delta_{\text{I}}$, $\Delta_{\text{X}}$, $\Delta_{\text{G}}$	Ultimate bounds of the sets D_I_, D_X_, D_G_
